# Risk of dementia associated with body mass index, changes in body weight and waist circumference in older people with type 2 diabetes: The Edinburgh Type 2 Diabetes Study

**DOI:** 10.1111/dme.15063

**Published:** 2023-03-03

**Authors:** Bo‐Jen Chen, Anniek J. Sluiman, Wardah Khalid, Mark W. J. Strachan, Jackie F. Price

**Affiliations:** ^1^ College of Medicine and Veterinary Medicine Usher Institute, University of Edinburgh Edinburgh UK; ^2^ Department of Internal Medicine National Taiwan University Hospital Taipei Taiwan; ^3^ Metabolic Unit Western General Hospital Edinburgh UK

**Keywords:** body mass index (BMI), body weight loss, dementia, type 2 diabetes, waist circumference

## Abstract

**Aims/Hypothesis:**

To determine the relationship of dementia with preceding body mass index (BMI), changes in body weight and waist circumference in older people with type 2 diabetes.

**Methods:**

In the Edinburgh Type 2 Diabetes Study (1064 men and women with type 2 diabetes, aged 60–75), body weight, waist circumference and BMI were measured at baseline and after 4 years in a subgroup (*n* = 821). Percentage body weight and waist circumference change over 4 years were calculated. Data on incident dementia was recorded during a median follow‐up time of 10.84 years. Survival models considering a range of co‐variables and/or death as a competing risk were used to estimate the risks of dementia associated with each weight‐related variable.

**Results:**

A total of 105 incident dementia events were recorded. When compared with people in the lowest BMI group (<25 kg/m^2^), risk of dementia was lower in intermediate BMI groups (25–29.9 kg/m^2^, HR 0.44, *p* = 0.002; 30–34.9 kg/m^2^, HR 0.41, *p* = 0.001) and the highest BMI group (≧35 kg/m^2^, HR 0.35, *p* = 0.001). In the weight change subgroup, 78 incident dementia events were recorded between years 4 and 10. Body weight loss over 5% (compared with ≦5%) was associated with higher incidence of dementia (HR 2.06, *p* = 0.010). The association between waist circumference change and dementia was not significant.

**Conclusions/Interpretations:**

Both a lower BMI and weight loss over a period of years are indicative of increased dementia risk for older people with type 2 diabetes, while waist circumference changes may be less informative.

**Novelty statement**

**What is already known?**

- Reduced body weight and waist circumference could be an early sign of dementia in older general populations, but their roles in type 2 diabetes populations are unclear.

**What this study has found?**

- Older people with type 2 diabetes experiencing major weight loss have a higher risk of developing dementia than those who maintain stable weight or gain weight.
- Changes in waist circumference do not seem to associate with subsequent dementia.

**What are the implications of the study?**

- When weight loss is observed in older people with type 2 diabetes, the possibility that this is an early sign of incident dementia should be considered.

## 1 INTRODUCTION

Considerable mortality and morbidity from type 2 diabetes and related comorbidities, including vascular disease and obesity, is well‐recognized.^1^ Type 2 diabetes populations also have a higher risk of Alzheimer's disease and vascular dementia compared with general population,^2^ and midlife obesity itself may be a risk factor for Alzheimer's disease.^3^ Clinically, intentional weight loss of at least 5% body weight has been recommended for obese individuals to improve insulin sensitivity and subsequent health outcomes.^4^ Conversely, unintentional weight loss may be a sign of underlying poor health,^5^ and in the general population, there is evidence that reduced body weight and waist circumference could be an early sign of senile dementia. For example, mean body mass index (BMI) and waist circumference (WC) are significantly lower from 6 to 8 years before a subsequent diagnosis of dementia.^6^, ^7^ A body shape index (ABSI), a newly developed marker, has also been proposed to predict premature mortality in the general population.^8^

In people with type 2 diabetes, there are limited studies addressing the relationship of obesity with dementia, and results are inconsistent. Some studies suggested that marked body weight loss or weight gain (10% or over) may be associated with an increased risk of dementia,^9^ while others failed to identify significant cognitive benefits or harm of weight loss during an intervention aimed at weight reduction amongst obese individuals with type 2 diabetes.^10^, ^11^ In the current study, we aimed to investigate the relationship of body weight and weight change, with incident dementia in older people with type 2 diabetes. We used data from the prospective Edinburgh Type 2 Diabetes Study, including long‐term follow‐up for dementia and serial measures of body weight and waist circumference from the earlier phases of the study.

## 2 METHODS

### 2.1 Study population

The Edinburgh Type 2 Diabetes Study (ET2DS) is a prospective cohort of men and women with type 2 diabetes. Full details of recruitment and participant examinations have been published previously.^12^

**FIGURE 1:**
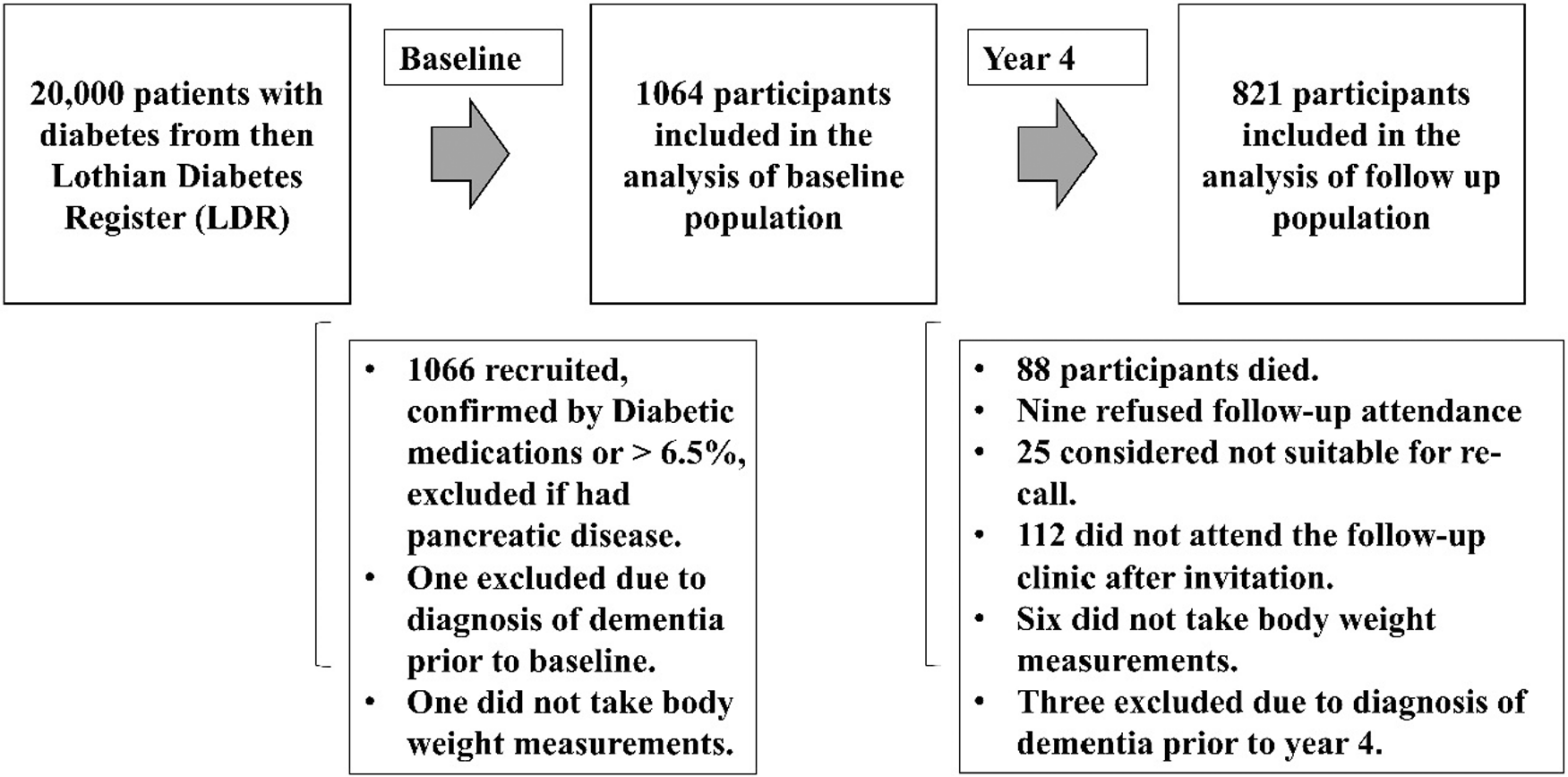
Consort diagram of the study population.

The study received full ethical approval from the Lothian Medical Research Ethics Committee and complies with the Declaration of Helsinki.^12^ The study population was selected at random, in 2006–2007, from the Lothian Diabetes Register (LDR), a database of around 20,000 patients with type 2 diabetes in total. The diagnosis of type 2 diabetes was confirmed if the individual was taking oral anti‐diabetic agents and/or insulin treatment or if HbA_1c_ level was over 6.5% and they did not have a history of pancreatic disease. All participants provided written informed consent. Individuals who were unable to receive physical examination or cognitive testing were excluded from the study. A total of 1066 participants were included in the baseline population, of whom, 1064 participants had body weight measurement taken and had not been diagnosed with dementia upon recruitment (Figure 1).

Year 4 follow‐up clinics for ET2DS participants were scheduled in 2010–2011. By this time, 88 (8.3%) participants had died, 9 (0.8%) refused follow‐up attendance, 25 (2.3%) were considered not suitable for re‐call and 112 (11%) did not attend the follow‐up clinic after invitation. This left 830 participants, of whom 824 had body weight measurements taken (Figure 1). The total baseline population (*n* = 1064) was used to explore the association of BMI with risk of dementia. The follow‐up population who had body weight measures available at both baseline and year four follow‐up was used to determine the association between change in body weight and risk of dementia. Three participants with a diagnosis of dementia prior to the year 4 follow‐up were excluded from the final analysis (*n* = 821).

### 2.2 Definition of dementia

The diagnosis of dementia was defined primarily using information from retrieval of medical records, which was carried out in February 2018, a median 10.84 years after baseline clinic. The criteria included (a) records from the National Health Service patient management system (TrakCare) searched from Nov 2017 to Feb 2018, (b) ICD‐10 codes from hospital discharge records, (c) death certificates codes searched in Feb 2018 and (d) prescribed medications for dementia (donepezil, rivastigmine, galantamine and memantine). At least two records of dementia from the above sources were required for the definition of dementia. An alternative was a dementia record from one source plus at least one supportive criterion, including (a) recorded results from mini‐mental state exam (MMSE) score ≤ 24, (b) reported dementia status from the participant's caregiver or (3) reported dementia from a general practitioner (GP). The criteria were developed by the ET2DS study team and have been reported previously.^13^

### 2.3 BMI, body weight and waist circumference variables

Weight‐related variables were derived using measurements taken at baseline and year 4.^12^ Height was measured to the nearest mm and weight to the nearest 0.1 kg. Waist circumference (WC) was measured between the lower rib margin and the iliac crest with the participant standing and exhaling. Measurements were conducted twice and the average to the nearest 0.5 cm was taken. BMI was calculated using the formula weight/height^2^ (kg/m^2^). According to The World Health Organization, obesity status was further categorized as underweight (BMI < 18.4 kg/m^2^), normal (18.5–25 kg/m^2^), overweight (25–29.9 kg/m^2^), Class I Obesity (30–34.9 kg/m^2^), class II or III obesity (BMI > 35 kg/m^2^).^14^ WC was categorized using quantiles. Weight change‐related variables, including % body weight (BW) change and WC change, were calculated using BW and WC data collected at baseline and year 4. When analysed as groups in survival models, BW change ≤5% was defined as “stable BW,” weight loss >5% was categorized into “major BW loss” and weight gain >5% into “major BW gain.” “Stable WC” was defined as ≤5 cm change in WC and an increase or decrease in WC >5 cm was defined as “major WC increase” or “major WC decrease,” respectively. ABSI was calculated with the formula WC/(BMI^2/3^ × height^1/2^).^8^

### 2.4 Co‐variables and cognitive test data

Descriptive and/or adjustment co‐variates included age, sex, education (highest education level reached as self‐reported in baseline health questionnaire), deprivation status (quintiles of Scottish Index of Multiple Deprivation (SIMD) with lower quintiles indicating higher deprivation), current treatment for type 2 diabetes (oral glucose‐lowering agents and/or insulin), and history of cardiovascular diseases (including myocardial infarction, angina, coronary or peripheral artery intervention, stroke, transient ischaemic attack and intermittent claudication), as well as cerebrovascular events (stroke or transient ischaemic attack) as defined previously.^15^ HbA_1c_, blood pressure, lipid profile (including serum total cholesterol and high‐density lipoprotein), creatinine and apolipoprotein E genotype were obtained from physiological measurements and venous blood sampling in the baseline study clinic. Self‐reported smoking and alcohol status (current drinking defined as any consumption in the year prior to completing the questionnaire) and lifetime history of hypoglycaemia events were collected at baseline by questionnaire. Smoking status was defined as current smoker, ex‐smoker or never smoker. A total of seven fluid‐type cognitive tests (Letter‐Number Sequencing (LNS), Borkowski Verbal Fluency Test (BVFT), Matrix Reasoning (MR), Digital Symbol Test (DST), Trial Making Test B (TMTB), Faces, Logical Memory (LM)) on various domains were also administered at both baseline and year 4. The date of any deaths was retrieved from death certificates stored in the National Records of Scotland.

### 2.5 Statistical analysis

Data analyses were conducted on R (version 3.6.3). Chi‐squared test for categorical variables and T test for continuous variables were calculated for differences between the population who developed dementia by the end of the study and those who did not. Similarly, chi‐squared tests or analysis of variance (ANOVA) were calculated between different groups of baseline obesity status, BW change or WC change groups.

Baseline BMI, WC, percentage BW change or WC change from baseline to year 4 were used as explanatory variables to predict outcome of incident dementia after year 4 using survival models. Univariable survival models using the Kaplan‐Meier method were used to present the probabilities of dementia‐free survival according to different percentage BW or WC change groups. Two models using cause‐specific cox proportional hazard function were conducted, adjusted for age and sex only or with additional covariables (baseline HbA_1c_, hypoglycaemic episodes, stroke, cardiovascular diseases, systolic blood pressure, total cholesterol, estimated glomerular filtration rate, education level, Scottish Index of Multiple Deprivation, smoking and alcohol consumption). An additional model using subdistribution hazard function was conducted, considering death as a competing risk in addition to end‐of‐study censoring. Additional survival models using cause‐specific cox proportional hazard function and subdistribution hazard function were also performed, adjusted for the covariables mentioned above and apolipoprotein E genotype. Samples with missing values in any of the covariables used were excluded from each of the models (Table S1). Cause‐specific hazard ratio (csHR) and subdistribution hazard ratio (sdHR) with Bonferroni‐adjusted 95% CI were calculated and presented for cause‐specific cox proportional hazard models and subdistribution hazard models, respectively.

In post‐hoc analysis, a derived factor “g” (used to reflect general cognitive function) was imputed from the seven fluid‐type cognitive tests applied at baseline using principle component analysis, as described previously.^16^ Multivariable linear regression models were used for the association between baseline cognitive function and BW change or WC change, and survival models were used for the association between cognitive function and dementia outcome.

## 3 RESULTS

### 3.1 Characteristics of the study population

Baseline characteristics of the ET2DS population without dementia at baseline (*n* = 1064), together with those of the subgroup (*n* = 821) used to assess the association between change in BW and dementia, are presented in Table 1. After a median follow‐up time of 10.84 years, 319 of 1064 participants had died, and 105 dementia events had occurred, which was in line with previously reported incidence (0.7% in all ages and approximately 1.5% in the 75–84 years old group).^17^ The median time‐to‐death from baseline was 6.38 years, and time‐to‐dementia was 7.10 years. In the subpopulation for addressing BW change (*n* = 821), 176 participants had died, and 78 events of incident dementia were recorded. Within this population, the median time‐to‐death was 4.24 years from year 4, and median time‐to‐dementia was 4.67. The majority of participants maintained stable BW (<5% change, 63%) or WC (<5 cm change, 59%) between baseline and year 4 follow‐up. The population had a relatively high socioeconomic status, with 43% having achieved highest levels of education (including professional or university degrees), and only 12% categorized in the most deprived quintile of Scottish Index of Multiple Deprivation (SIMD). The population had a mean HbA_1c_ controlled at 7.41 ± 1.13 (%). Half of the population had a recorded history of cardiovascular disease (50%), including 6% of the participants with stroke.

**TABLE 1:** Baseline characteristics of the total study population (*n* = 1064)^a^ and the subpopulation with body weight data available at both baseline and year 4 (*n* = 821), stratified by presence or absence of dementia by the end of follow‐up.

	Baseline			Year‐4		
	No dementia (*n* = 959)	Dementia (*n* = 105)	*p*‐value^b^	No dementia (*n* = 743)	Dementia (*n* = 78)	*p*‐value^b^
Age (years)	67.7 ± 4.2	69.4 ± 4.0	<0.001	67.5 ± 4.2	69.0 ± 3.9	0.002
Sex			0.048			0.127
Female	50 (477)	39 (41)		50 (368)	40 (31)	
Male	50 (482)	61 (64)		50 (375)	60 (47)	
Education			0.038			0.088
Primary school	1 (5)	2 (2)		1 (4)	1 (1)	
Secondary school	54 (516)	62 (65)		52 (388)	62 (48)	
Professional/Technical	30 (285)	19 (20)		30 (221)	18 (14)	
University/college	16 (153)	17 (18)		17 (130)	19 (15)	
SIMD Quintile			0.214			0.509
1	12 (114)	12 (13)		12 (89)	10 (8)	
2	20 (191)	16 (17)		18 (131)	14 (11)	
3	18 (168)	19 (20)		17 (123)	21 (16)	
4	17 (166)	26 (27)		17 (126)	23 (18)	
5	33 (320)	27 (28)		37 (274)	32 (25)	
Status of diabetes						
Duration of diabetes (years)	8.0 ± 6.4	9.0 ± 7.1	0.176	7.9 ± 6.4	8.8 ± 6.4	0.234
HbA_1c_ (mmol/mmol)	57.3 ± 11.7	58.9 ± 16.9	0.368	57.0 ± 11.7	60.3 ± 17.9	0.117
HbA_1c_ (%)	7.4 ± 1.1	7.5 ± 1.5	0.368	7.4 ± 1.1	7.7 ± 1.6	0.117
Treatment of diabetes			0.606			0.398
Diet‐controlled	19 (180)	17 (18)		20 (147)	17 (13)	
Oral medication	64 (614)	62 (65)		64 (476)	62 (48)	
Insulin injections	17 (164)	21 (22)		16 (119)	22 (17)	
Weight related variables						
Height (cm)	166.0 ± 9.2	166.3 ± 9.5	0.762	166.2 ± 8.9	166.2 ± 9.0	0.997
Weight (kg)	86.9 ± 16.2	82.9 ± 15. 8	0.018	86.6 ± 16.0	83.5 ± 15.8	0.112
BMI (kg/m2)	31.6 ± 5.7	30.0 ± 5.2	0.004	31.4 ± 5.6	30.2 ± 5.2	0.067
<18.4	0 (1)	0 (0)	0.018	0 (1)	0 (0)	0.214
18.5–25	9 (85)	18 (19)		9 (65)	15 (12)	
25–29.9	34 (324)	37 (39)		36 (266)	37 (29)	
30–34.9	33 (321)	30 (31)		33 (247)	33 (26)	
>35	24 (228)	15 (16)		22 (164)	14 (11)	
Waist (cm)^c^	107.2 ± 12.8	104.5 ± 13.0	0.046	106.66 ± 12.54	105.16 ± 12.78	0.323
Q1	24 (228)	30 (32)	0.287	24 (182)	29 (23)	0.812
Q2	25 (236)	28 (29)		26 (196)	26 (20)	
Q3	26 (245)	22 (23)		26 (194)	24 (19)	
Q4	26 (246)	20 (21)		23 (169)	21 (16)	
Hip (cm)	111.4 ± 12.2	106.7 ± 9.3	<0.001	111.0 ± 12.0	106.8 ± 8.8	<0.001
WHR	0.96 ± 0.07	0.98 ± 0.08	0.079	0.96 ± 0.07	0.98 ± 0.08	0.027
Other metabolic risk factors						
Systolic blood pressure (mm Hg)	133 ± 17	134 ± 16	0.578	133 ± 16	133 ± 14	0.878
Diastolic blood pressure (mm Hg)	69 ± 9	69 ± 10	0.830	69.0 ± 8.7	69.2 ± 9.7	
Total Cholesterol (mmol/L)	4.3 ± 0.9	4.2 ± 1.0	0.434	4.4 ± 0.9	4.2 ± 0.9	0.303
eGFR (ml/min)	64.4 ± 14.8	64.8 ± 14.1	0.783	64.9 ± 14.4	65.1 ± 15.1	0.910
Medical history						
Cardiovascular disease^d^			0.112			0.076
No	51 (488)	42 (44)		54 (401)	43 (33)	
Yes	49 (468)	57 (60)		46 (339)	57 (44)	
Stroke			0.832			0.789
No	94 (905)	93 (98)		95 (704)	96 (75)	
Yes	6 (54)	7 (7)		5 (39)	4 (3)	
Hypoglycaemic attack			0.251			0.089
No	88 (841)	83 (87)		90 (666)	83 (65)	
Yes	10 (98)	14 (15)		9 (65)	15 (12)	
Lifestyle factors						
Smoker			0.120			0.323
Never	39 (377)	35 (37)		40 (299)	44 (34)	
Former	46 (438)	55 (58)		46 (342)	49 (38)	
Current	15 (144)	10 (10)		14 (102)	8 (6)	
Current alcohol drinkers			1.000			0.812
No	19 (184)	19 (20)		16 (120)	18 (14)	
Yes	80 (771)	80 (84)		84 (621)	82 (64)	
Genetic factor						
Apolipoprotein E (APOE) genotype			<0.001			<0.001
ε2/ε2 or ε2/ε3 or ε3/ε3	72 (693)	48 (50)		73 (545)	47 (37)	
ε2/ε4 or ε3/ε4	22 (213)	43 (45)		21 (159)	42 (33)	
ε4/ε4	1 (13)	7 (7)		2 (12)	6 (5)	

*Note*: Values are presented as Mean ± SD, median (Q1, Q3) or % (*n*).

Abbreviations: DBP, diastolic blood pressure; eGFR, estimated Glomerular Filtration Rate; SBP, systolic blood pressure; SIMD: Scottish Index of Multiple Deprivation 2006 (quintile 1 is most deprived); WHR, waist–hip ratio.

^a^ Of 1066 participants in the ET2DS, two individuals were excluded from the current analyses due to pre‐existing dementia and missing body weight measurements at baseline, respectively.

^b^ Calculated by *t*‐test (for numerical variables), chi‐squared test (for categorical variables) or Fisher's exact test (for BMI groups).

^c^ Quantiles of baseline waist circumference in the total population (male/female) (cm): Q1: 78–99.9/73–95.9, Q2: 100–106.4/96–104.9, Q3: 106.5–115.9/105–113.8, Q4: 116–153.9/113.9–158.9, in the population with year 4 follow up: Q1: 83–99.7/78–91.1, Q2: 99.8–106.4/91.2–100.9, Q3: 106.5–113.4/101–110.6, Q4: 113.5–139.3/110.7–126.

^d^ Cardiovascular disease includes established history of intermittent claudication, myocardial infarction, angina, transient ischaemic attack, coronary or peripheral artery intervention or ankle brachial pressure index below 0.9.

### 3.2 Dementia risk according to baseline BMI and WC

The risk of dementia associated with baseline body weight, BMI and WC is presented in Table 2. The underweight group was excluded from the models as only one participant fell into this category. Risk of dementia was significantly lower in groups with overweight (csHR 0.44, 95% CI 0.25–0.79, *p* = 0.006), class I obesity (csHR 0.41, 95% CI 0.22–0.75, *p* = 0.004) and class II/III obesity (csHR 0.35; 95% CI 0.17–0.71, *p* = 0.004) when compared with the normal BMI group, after adjustment for age, sex, HbA_1c_, history of hypoglycaemic episodes, stroke, cardiovascular disease, systolic blood pressure, total cholesterol, educational attainment, SIMD, smoking and alcohol consumption. The trend was still present when using competing risk subdistribution multivariable hazard model. In contrast, the risk of dementia associated with WC or ABSI at baseline did not reach statistical significance.

**TABLE 2:** Risk of dementia associated with baseline body weight, BMI and waist circumference.

	Median time to dementia (years)	Model 1^a^		Model 2^b^		Model 3^c^	
		csHR (95% CI compared to reference)	*p*‐value	csHR (95% CI compared to reference)	*p*‐value	csHR (95% CI compared to reference)	*p*‐value
Body Mass Index (WHO classifications)							
Per 5 kg/m^2^ increase		0.86 (0.70–1.05)	0.135	0.83 (0.67–1.04)	0.103	0.82 (0.65–1.03)	0.093
Normal	5.85	1 (Ref.)		1 (Ref.)		1 (ref.)	
Overweight	6.55	0.54 (0.31–0.93)	0.027	0.44 (0.25–0.79)	0.006	0.47 (0.26–0.85)	0.012
Obesity I	8.55	0.52 (0.30–0.93)	0.027	0.41 (0.22–0.75)	0.004	0.43 (0.23–0.79)	0.006
Obesity II/III	7.08	0.46 (0.23–0.89)	0.022	0.35 (0.17–0.72)	0.004	0.35 (0.17–0.72)	0.005
Waist Circumference (quantiles)^d^							
Per 5 cm increase		0.95 (0.87–1.03)	0.185	0.94 (0.86–1.03)	0.161	0.93 (0.85–1.01)	0.097
Q1	6.67	1 (Ref.)		1 (Ref.)		1 (Ref.)	
Q2	7.53	0.92 (0.56–1.53)	0.761	0.95 (0.56–1.62)	0.851	0.94 (0.55–1.60)	0.810
Q3	7.92	0.78 (0.46–1.34)	0.371	0.75 (0.42–1.34)	0.335	0.74 (0.42–1.30)	0.290
Q4	6.92	0.84 (0.48–1.47)	0.543	0.78 (0.43–1.41)	0.407	0.70 (0.39–1.25)	0.230
ABSI (per 0.01 increase)		2.07 (0.80–5.35)	0.135	1.09 (0.65–1.83)	0.749	0.98 (0.58–1.68)	0.950

Abbreviations: csHR, cause‐specific hazard ratio; Ref., reference; sdHR, subdistribution hazard ratio.

^a^ Model 1, Cox proportional hazard multivariable model, adjusted for age and sex. Maximum total number = 1064 (after excluding two samples with pre‐existing dementia and missing body weight measurements from the ET2DS).

^b^ Model 2, Cox proportional hazard multivariable model, adjusted for age, sex, baseline HbA_1c_, history of hypoglycaemic episodes, history of stroke, history of cardiovascular diseases, systolic blood pressure, total cholesterol, Estimated Glomerular Filtration Rate (eGFR), education attenuation, Scottish Index of Multiple Deprivation, smoker and alcohol drinker. Maximum total number = 1010.

^c^ Model 3, Competing risks subdistribution multivariable hazard model, adjusted for age, sex, baseline HbA_1c_, history of hypoglycaemic episodes, history of stroke, history of cardiovascular diseases, systolic blood pressure, total cholesterol, estimated Glomerular Filtration Rate, education attenuation, Scottish Index of Multiple Deprivation, smoker and alcohol drinker. Maximum total number = 1010.

^d^ Quantiles of waist circumference: Q1: 83–99.7/78–91.1, Q2: 99.8–106.4/91.2–100.9, Q3: 106.5–113.4/101–110.6, Q4: 113.5–139.3/110.7–126.

### 3.3 Dementia risk according to BW and WC change

**FIGURE 2:**
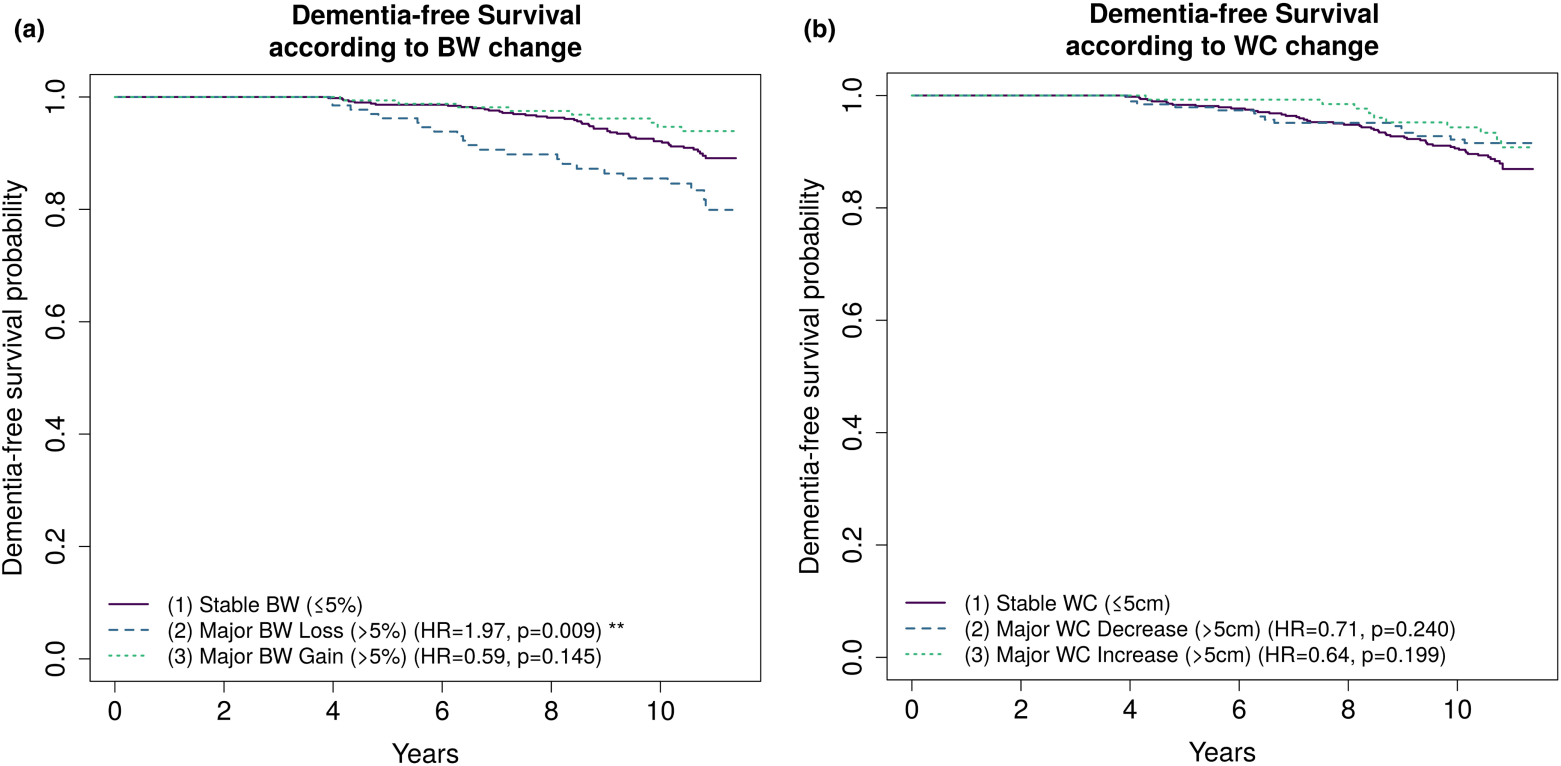
Kaplan–Meier curves of dementia‐free survival according to changes in BW or WC. (a) Dementia‐free survival according to BW change groups (1) Stable BW (≤5%) (ref.), (2) Major BW loss (>5%) (HR = 1.97, *p* = 0.009), (3) Major BW gain (>5%) (HR = 0.59, *p* = 0.145). (b) Dementia‐free survival according to WC change groups (1) stable WC (≤5 cm) (ref.), (2) MAJOR WC decrease (>5 cm) (HR = 0.71, *p* = 0.240), (3) Major WC increase (>5 cm) (HR = 0.64, *p* = 0.199). BW, body weight, WC, waist circumference.

The risk of dementia associated with BW change and WC change is presented in Table 3 (cox‐proportional hazard models and competing risk subdistribution multivariable hazard models) and Figure 2 (Kaplan Meier curves). Individuals who experienced major BW loss from baseline to year 4, compared with their stable BW counterparts, were significantly more likely to develop dementia. The csHR in cox proportional hazard model was 2.19 (*p* = 0.003) after adjustment for age and sex and 2.09 (*p* = 0.008) after full multi‐variable adjustment. The association of weight loss with higher risk of dementia persisted in the subdistribution hazard model accounting for competing risk of death (BW loss group compared with stable BW group, sdHR 2.09, *p* = 0.013). When further adjusted for Apolipoprotein E genotype, the sdHR slightly attenuated and the p‐value became borderline non‐significant (BW loss group compared with stable BW group, sdHR 1.81, *p* = 0.068). (Table S2). Changes in WC were not statistically significantly associated with risk of dementia.

**TABLE 3:** Risk of dementia associated with changes in obesity‐related physiological factors.

	Dementia events (%)	Median time to dementia (years)	Model 1^a^		Model 2^b^		Model 3^c^	
			csHR (95% CI compared to reference)	*p*‐value	csHR (95% CI compared to reference)	*p*‐value	csHR (95% CI compared to reference)	*p*‐value
Body weight (BW) change								
Per 5% BW increase			0.97 (0.93–1.00)	0.046	0.96 (0.93–1.00)	0.044	0.96 (0.92–1.01)	0.140
Major BW loss	22 (17)	2.75	2.19 (1.31–3.65)	0.003	2.09 (1.21–3.62)	0.008	2.09 (1.17–3.74)	0.013
Stable BW	47 (9.1)	4.87	1 (ref.)		1 (ref.)		1 (ref.)	
Major BW gain	9 (5.4)	4.76	0.63 (0.31–1.28)	0.198	0.56 (0.27–1.16)	0.119	0.54 (0.26–1.14)	0.110
Waist circumference (WC) change								
Per 5 cm WC increase			0.98 (0.95–1.01)	0.185	0.98 (0.95–1.02)	0.298	0.98 (0.94–1.02)	0.270
Major WC decrease	15 (7.8)	2.75	0.74 (0.42–1.31)	0.300	0.63 (0.33–1.19)	0.157	0.64 (0.33–1.22)	0.180
Stable WC	53 (11)	4.62	1 (ref.)		1 (ref.)		1 (ref.)	
Major WC increase	10 (7.2)	4.88	0.64 (0.33–1.26)	0.197	0.63 (0.32–1.26)	0.190	0.63 (0.32–1.26)	0.190

Abbreviations: csHR, cause‐specific hazard ratio; Ref., reference; sdHR, subdistribution hazard ratio.

^a^ Model 1, Cox proportional hazard multivariable model, adjusted for age and sex. Maximum total number = 821.

^b^ Model 2, Cox proportional hazard multivariable model, adjusted for age, sex, baseline HbA_1c_, history of hypoglycaemic episodes, history of stroke, history of cardiovascular diseases, systolic blood pressure, total cholesterol, estimated Glomerular Filtration Rate, education attenuation, Scottish Index of Multiple Deprivation, smoker and alcohol drinker. Maximum total number = 780.

^c^ Model 3, Competing risks subdistribution multivariable hazard model, adjusted for age, sex, baseline HbA_1c_, history of hypoglycaemic episodes, history of stroke, history of cardiovascular diseases, systolic blood pressure, total cholesterol, education attenuation, Scottish Index of Multiple Deprivation, smoker, alcohol drinker and baseline body mass index for body weight change or baseline waist circumference for waist circumference change. Maximum total number = 780.

### 3.4 Association of BW change and WC change with baseline cognitive ability (post‐hoc analysis)

A measure of general cognitive ability “g” (derived from a cognitive test battery applied at baseline) was positively associated with subsequent change in BW (*β* = 0.061, *p* = 0.016) and WC (*β* = 0.081, *p* = 0.003). Lower baseline “g” was also associated with higher incidence of dementia. Additional survival models for the association between BW change and dementia, adjusted for the covariables listed above as well as baseline “g” were performed, which led to a non‐significant p‐value in the association between BW change and dementia (Cox proportional hazard model, BW loss group compared with stable BW group, csHR 1.71, *p* = 0.085).

## 4 DISCUSSION

In the current study on older people with type 2 diabetes, BW loss over 5% was found to be significantly associated with a higher risk of subsequent dementia compared with maintenance of a stable BW. This association persisted after adjustment for multiple cardiovascular and diabetes‐related risk factors. There was no evidence of association between BW gain and risk of dementia, and changes in WC were not significantly associated with dementia. Poorer cognitive function at baseline was associated with both subsequent BW loss and incident dementia.

The potential role of BW loss as an early indicator of dementia has been reported in some previous studies. In general populations, significantly lower BMI was observed from 6 to 8 years prior to diagnosis of dementia,^5^, ^6^ which was consistent with a negative association between baseline BMI and incidence of dementia in our study (mean time‐to‐dementia 7.08 years). BW loss has been suggested previously to be associated with dementia in type 2 diabetes populations but evidence is limited. A “U‐shaped” association was observed among patients with type 2 diabetes, with >10% weight gain, as well as >10% weight loss within 2 years associated with increased dementia incidence.^9^ However, as weight gain over 10% is rare in older populations (comprising only 4% (*n* = 26) of our study population), we were unable to assess the incidence of dementia in this subpopulation in our study. There are further inconsistencies in the results from interventional studies, possibly due to younger mean ages at baseline and higher proportion of intentional weight loss in such populations. A lifestyle interventional study in people with impaired glucose tolerance found that participants experiencing prolonged weight loss (from the interventional phase to an extended follow‐up phase) had the worst cognitive outcomes.^18^ In contrast, the association between weight loss and cognitive function was not found to be significant in another clinical trial aimed at weight loss induced by intensive lifestyle intervention in participants with type 2 diabetes.^10^, ^11^

In our study, unlike the significant association between BW loss and increased dementia risk, major WC decrease showed a trend for association with a lower risk of dementia which did not reach statistical significance. This was different from patterns of WC decrease and BW loss preceding dementia diagnosis found in previous studies on the general population.^7^ An observed association of dementia with reduction in BW, but not WC, may represent a status of sarcopenia. Poor nutrition, which is a common feature in patients with dementia, together with physical inactivity, is thought to form a vicious cycle leading to sarcopenia, with insulin resistance playing an important role.^19^, ^20^ This was compatible with older subjects with type 2 diabetes being found to have a higher risk of sarcopenia than their non‐diabetic counterparts, despite having a higher BMI in general.^21^ Thus, people with type 2 diabetes are potentially more prone to muscular tissue loss related to physical inactivity and poor nutrition status caused by cognitive dysfunction. Although the total sample size in our study was insufficient to analyse the inconsistent presentation of physiological change at an individual level (the number of participants fulfilling changes in BW but not in WC, or vice versa, was insufficient for grouped analysis), such a grouping approach should be considered in larger, future studies, to explore this issue further.

Our study has some limitations. As well as limited sample size to explore associations across all possible groupings (including weight loss or weight gain over 10%, and different aetiologies of dementia, including Alzheimer's disease or vascular dementia), there is likely some selection bias in favour of those participants who attended the year 4 follow up clinic. Missing diagnoses of dementia is also possible, although this was minimized by comprehensive searches of multiple data sources for possible cases. For example, although all participants had records on the National Health Service patient management system (TrakCare), covering routine healthcare visits, and were further followed by national record linkage to hospital discharge data, it is possible that incident dementia may have occurred in the very small number of participants who moved away from Scotland. The csHR of BW change associated with dementia was no longer significant after adjustment for apolipoprotein E genotype and baseline cognitive function in the fully adjusted model (both of which were significantly associated with risk of dementia), which was likely affected by the reduction in sample size due to a larger number of missing values for apolipoprotein E genotype (*n* = 30) and baseline cognitive function (*n* = 25). Although carefully adjusted for multiple, pre‐selected covariables, given the complexity of the pathophysiological mechanisms underlying type 2 diabetes, obesity and dementia, and the sharing of many risk factors, there is the possibility of either over‐adjustment or lack of adjustment for true and/or fully representative confounders. Only binary variables created from self‐reported information were used for alcohol drinking and hypoglycaemia events. Also, due to limited data on lifestyle habits, such as food intake and exercise, we were unable to explore possible underlying causes of weight change in the analysis, such as intentional weight loss. Similarly, cancer (which may potentially lead to unintentional weight loss) was not considered in our analyses, and we also did not have sufficient data to define sarcopenia status at baseline to be able to explore the possible role of this condition in our findings. Lastly, the model including BW change as a continuous variable, but not as grouped variables, violated the proportional hazard assumption. A time‐dependent effect of BW change on dementia is possible. The exact effect of time on the relationship between BW change and dementia requires further investigation.

Of note, the medications for type 2 diabetes which the participants were taking at baseline in this study were either metformin, sulfonylureas, thiazolidinediones or insulin (with an exception of only 2 participants taking alpha‐glucosidase inhibitors). Currently, people with type 2 diabetes are increasingly prescribed with newly introduced oral glucose‐lowering agents, such as sodium glucose co‐transporter 2 inhibitors (SGLT2 Is), glucagon‐like peptide‐1 receptor agonists (GLP‐1 RAs) or dipeptidyl peptidase‐4 inhibitors (DPP4 Is). Given that metformin, SGLT2 Is, GLP‐1 RAs and alpha‐glucosidase inhibitors may be beneficial for weight loss, while sulfonylureas, thiazolidinediones and insulin may be associated with weight gain, weight loss observed in type 2 diabetes patients nowadays are more likely to result from medication.^22^ Similarly, as promotion of weight loss is more frequently introduced in clinical practice, the proportion of “unintentional” weight loss in our study population may differ from the general type 2 diabetes populations nowadays. Nevertheless, this study is the first to comprehensively address BW change, WC change, dementia, and cognitive function change in a single type 2 diabetes population. It benefits from presenting both cause‐specific cox proportional hazard models, optimal for investigating etiologic questions, and subdistribution hazard models, considering death as a competing risk. The latter were recommended in older populations to reduce possible overestimation of outcome variables in the former models, which showed similar directions of association with only slightly reduced significance.^23^

In conclusion, this longitudinal study suggested that body weight loss is associated with increased risk of dementia in older people with type 2 diabetes. The association between poorer baseline cognitive function and subsequent weight loss favours weight loss being an early sign of dementia in people with type 2 diabetes. When physicians observe weight loss in older type 2 diabetes patients, particularly in the absence of intentional weight loss, effect of medications or other secondary causes, the possibility that this is an early sign of incident dementia should be considered.

## AUTHOR CONTRIBUTIONs

Bo‐Jen Chen performed the data analysis and drafted the manuscript under supervision from Jackie F. Price. Anniek J. Sluiman contributed to data collection, data analysis, interpretation of findings and preparation of the manuscript. Wardah Khalid and Mark W. J. Strachan contributed to data collection, interpretation of findings and preparation of the final manuscript. Jackie F. Price contributed to the interpretation of findings and preparation of the final manuscript, conceived the design of the ET2DS and oversaw the data acquisition and analysis. All authors read and revised the manuscript and approved the final version. Jackie F. Price is the guarantor of this work.

## FUNDING INFORMATION

The ET2DS was sponsored by the University of Edinburgh. The study was funded by the Medical Research Council (UK) (ProjectGrant G0500877), the Chief Scientist Office of the Scottish Executive (Programme Support Grant CZQ/1/38), Pfizer plc. and DiabetesUK (Clinical Research Fellowship 10/0003985). The study sponsor/funder was not involved in the design of the study; the collection, analysis, and interpretation of data; writing the report; and did not impose any restrictions regarding the publication of the report.

## CONFLICT OF INTEREST STATEMENT

The authors declare that there are no relationships or activities that might bias, or be perceived to bias, their work.

## Supporting information


Appendix S1.

## Data Availability

The datasets analysed during the current study are available from the corresponding author on reasonable request.
