# Supplementary material for: Risk of dementia associated with body mass index, changes in body weight and waist circumference in older people with type 2 diabetes: The Edinburgh Type 2 Diabetes Study
Source: Diabet Med. 2023 Mar 3;40(5):e15063. doi: 10.1111/dme.15063 (PMC10947035; doi:10.1111/dme.15063)
Supplement: Supplementary file 1 — Appendix S1. [file DME-40-0-s001.docx]

Table 1. Missing data for variables included in analyses from baseline and 4-year follow-up datasets

|  | Missing data at baseline | Missing data at year-4 follow up |
| --- | --- | --- |
| Age | 0 | 0 |
| Sex | 0 | 0 |
| Education | 0 | 0 |
| SIMD Quintile | 0 | 0 |
| Duration of diabetes | 13 | 7 |
| HbA1c | 9 | 6 |
| Treatment of diabetes | 1 | 1 |
| Height | 0 | 0 |
| Weight | 0 | 0 |
| BMI | 0 | 0 |
| Waist | 4 | 2 |
| Hip | 4 | 2 |
| Waist hip ratio | 5 | 2 |
| Systolic blood pressure | 2 | 2 |
| Diastolic blood pressure | 2 | 2 |
| Total Cholesterol | 9 | 5 |
| Estimated Glomerular Filtration Rate | 13 | 8 |
| Cardiovascular disease * | 4 | 4 |
| Stroke | 0 | 0 |
| Hypoglycemic attack | 23 | 14 |
| Smoker | 0 | 0 |
| Current Alcohol Drinkers | 5 | 2 |
| Apolipoprotein E (APOE) genotype | 43 | 30 |

SIMD: Scottish Index of Multiple Deprivation 2006

Table 2. Risk of dementia associated with changes in obesity related physiological factors

|  | Median time to dementia (years) | csHR (95% CI, p-value compared to reference) | | sdHR (95% CI, p-value compared to reference) |  |
| --- | --- | --- | --- | --- | --- |
|  |  | Model 1 ^a^ | Model 2 ^b^ | Model 3 ^c^ |  |
| Body weight (BW) change |  |  |  |  |  |
| Per 5% BW increase |  | 0.97 (0.93-1.00, p=0.046) | 0.97 (0.94-1.01, p=0.138) | 0.97 (0.93-1.02, p=0.280) |  |
| Major BW loss | 2.75 | 2.19 (1.31-3.65, p=0.003) | 1.87 (1.03-3.42, p=0.041) | 1.81 (0.96-3.41, p=0.068) |  |
| Stable BW | 4.87 | 1 (ref.) | 1 (ref.) | 1 (ref.) |  |
| Major BW gain | 4.76 | 0.63 (0.31-1.28, p=0.198) | 0.62 (0.29-1.33, p=0.221) | 0.59 (0.26-1.32, p=0.200) |  |
| Waist circumference (WC) change |  |  |  |  |  |
| Per 5 cm WC increase |  | 0.98 (0.95-1.01, p=0.185) | 0.99 (0.97-1.01, p=0.203) | 0.98 (0.96-1.01, p=0.170) |  |
| Major WC decrease | 2.75 | 0.74 (0.42-1.31, p=0.300) | 0.52 (0.26-1.04, p=0.065) | 0.52 (0.26-1.04, p=0.063) |  |
| Stable WC | 4.62 | 1 (ref.) | 1 (ref.) | 1 (ref.) |  |
| Major WC increase | 4.88 | 0.64 (0.33-1.26, p=0.197) | 0.63 (0.31-1.26, p=0.189) | 0.62 (0.32-1.23, p=0.170) |  |

Ref., reference. csHR, cause-specific hazard ratio, sdHR, subdistribution hazard ratio. ^a^ Model 1, Cox proportional hazard multivariable model, adjusted for age and sex. Maximum total number =821. ^b^ Model 2, Cox proportional hazard multivariable model, adjusted for age, sex, baseline HbA1c, history of hypoglycemic episodes, history of stroke, history of cardiovascular diseases, systolic blood pressure, total cholesterol, estimated Glomerular Filtration Rate, education attenuation, Scottish Index of Multiple Deprivation, smoker, alcohol drinker and Apolipoprotein E genotype. Maximum total number =752. ^c^ Model 3, Competing risks sub-distribution multivariable hazard model, adjusted for age, sex, baseline HbA1c, history of hypoglycemic episodes, history of stroke, history of cardiovascular diseases, systolic blood pressure, total cholesterol, education attenuation, Scottish Index of Multiple Deprivation, smoker, alcohol drinker, Apolipoprotein E genotype and baseline body mass index for body weight change or baseline waist circumference for waist circumference change. Maximum total number =752.
